# Unnatural Death among Treatment Seeking Substance Users in Singapore: A Retrospective Study

**DOI:** 10.3390/ijerph16152743

**Published:** 2019-07-31

**Authors:** Asharani PV, Tan Jun Wen, Mohamed Zakir Karuvetil, Alvin Cheong, Christopher Cheok, Gomathinayagam Kandasami

**Affiliations:** 1 Research Division, Institute of Mental Health, 10 Buangkok View, Singapore 539747, Singapore; 2 National Addictions Management Service, Institute of Mental Health, 10 Buangkok View, Singapore 539747, Singapore; 3 Forensic Psychiatry, Institute of Mental Health, 10 Buangkok View, Singapore 539747, Singapore

**Keywords:** unnatural death, suicide, accidental death, overdose, opiates, alcohol, substance use

## Abstract

Substance use carries a higher risk of unnatural death. A retrospective analysis of 42 treatment seekers between 2011–2015 was conducted through medical record review to understand the profile and circumstances leading to premature deaths. Ninety percent of the subjects were males. The mean age at death was 44.9 (SD ± 13.1). Opioids (52.4%), benzodiazepines (45.2%) and alcohol (35.7%) were the main substances used by the group. Suicide was the most common cause of death (*n* = 27, 64.3%) followed by accidents (*n* = 15; 35.7%). Among the suicide cases, alcohol was the main substance used (33.3%) followed by opioids (25.9%). A total of 58.5% (*n* = 24) deaths occurred within a year of their last visit while 41.5% (*n* = 17) were dead more than a year after their last visit. Of the total cases (*n* = 41), 63% had a history of mental illness with depressive disorder (53.8%) being the most common. History of suicide attempts were reported in 34.1% (*n* = 14) of cases and 50% of the subjects (*n* = 21) had a history of suicidal ideation. Drug related offences were reported in 57.1% of the subjects, of which 60% (*n* = 18) committed suicide. The findings support the need for appropriate treatment resources to reduce the untimely deaths among substance users.

## 1. Introduction

Unnatural deaths are preventable mortality, the number and frequency of which reflects the quality and effectiveness of public health services. Whilst natural deaths occur through disease progression, unnatural deaths result from unnatural or external causes that include suicide, homicide and accidents. Substance use and mental illness are major risk factors for unnatural deaths [1,2,3,4].

The type and number of substances used are key contributors to unnatural deaths. Approximately 450,000 drug use related deaths were reported globally in 2015, with opiate use accounting for 76% of these deaths [5]. A systematic review and meta-analysis showed that the risk of death increases by 15 fold (crude mortality ratio 2.09 per 100 person years) in opiate users [6]. Mortality due to overdose was reported more frequently than other causes. A study comparing the mortality ratios and expected years of life lost among two cohorts—opioid-dependent subjects from the U.S. and Taiwan—showed that half of the subjects died of unnatural causes in both cohorts, with overdose being the common cause of death in the U.S. cohort (U.S.: 80.6%, Taiwan: 25%) as compared to suicide in the Taiwan cohort (Taiwan: 51.9%, U.S.: 2.8%). [7]. Similarly, other substances are also implicated in unnatural deaths. A study showed that amphetamine users had an excessive mortality due to unnatural deaths with a standardised mortality rate of 6.02 [8]. Alcohol plays a causal role in unnatural deaths [9,10,11], which includes deaths due to respiratory depression [12]. Cognitive impairments resulting from chronic alcohol abuse have been linked to accidents [13,14], falls [15,16], drowning [17], suicide [18,19] and homicide [20].

Number of substances used are also important determinants of mortality. Bradvick and colleagues [21] studied the relationship between number of addictive substances used and the risk of unnatural death. The study showed a higher risk with every additional substance abused. A study conducted in Hungary reported heroin either alone (58%) or in combination with alcohol (26%) played a causal role in drug overdose deaths [22]. Tuusov et al. [23] analysed nine years of data on unnatural death among substance users and reported ethanol poisoning (35.1%) as the main cause of death followed by carbon monoxide (27.9%) and illegal drugs (21.5%).

Suicides and accidents are commonly cited causes of death among substance users [24,25]. Lee et al. [26] studied drug related deaths among a cohort of substance users and showed a 57% increase in drug related deaths between 2001 to 2013, of which 81.6% were unnatural deaths exhibiting distinctive demographic profiles for individual drug types and death categories (accidents, suicides, homicides, etc.). Several meta-analysis have shown higher odds of suicides, suicide ideations and suicide attempts in substance users [27,28].

In Singapore, 51.07% of the cases between 2009 and 2010 [29] reported to the coroner were unnatural deaths which included drug toxicity/adverse reactions (6.93%). Falls from height (35.86%), accidents (17.6%) and drowning (5%) were also reported, however, the number of substance users among the cases was unknown. Understanding the substance user profile, habits and cause of death is necessary to implement relevant preventive programmes. This study was mainly aimed at understanding the specific demographics, substance user profiles and circumstances leading to unnatural deaths among our clinical population to allow the clinicians to be aware of such risks and implement key preventative measures to minimise the risk.

## 2. Materials and Methods

### 2.1. Study Population

In Singapore, a case receives coroner’s attention when the cause of death is either unknown or suspicious. Coroner’s enquiry involves obtaining the medical report from the respective hospitals. National Addictions Management Service (NAMS) is the main provider of addiction treatment at the national level and hence it is also the recipient of such an enquiry when substance use is suspected and the deceased had sought treatment at NAMS. An internal database was created in 2011 to track the coroner’s enquiries. The study team compiled a list of such enquires between the period of 2011–2015 for this study. A retrospective analysis of this data was performed to ascertain the cause of death, circumstances of death, substance use trends and the clinical/ demographic profile of the patients. Cases of natural death, missing records and behavioral addictions were excluded from the study while cases of unnatural deaths or deaths due to undetermined causes where substance use was involved were included.

### 2.2. Data Collections

A total of 65 cases of unnatural deaths were identified during the study period. Death certificates were obtained from the Registry of Birth and Death, Immigration and Checkpoint Authority of Singapore (ICA). The cases were examined for cause of death. For cases where the cause of death was broad or unclear (e.g., cause of death indicated as multiple injuries, which can be a result of suicide (jumping from high rise buildings) or accidents), the Coroner’s verdict was collected from the State Courts, Singapore. The Coroner’s verdict includes a detailed description of the investigation and the final cause of death. Demographic (age, gender, race, education, etc.) and clinical data (Diagnostic and Statistical Manual of Mental Disorders, 4th Edition (DSM-IV) diagnosis, forensic history, engagement with treatment and community rehabilitation services, type, number of substances used, adverse childhood experiences, psychiatric history, suicide and self-harm history, etc.) were collected from patient’s medical records. The data was extracted by trained clinicians using a data collection form. Those with known substance use were included in the analysis. The deaths were broadly classified as natural deaths and unnatural deaths. Unnatural deaths were further classified as suicide, accidents and homicides. Twenty-three cases were excluded from the analysis, which included cases of natural deaths (15 cases), unnatural deaths related to gambling addiction, large amount of missing data and untraceable medical records. The study methodology was approved by the Domain Specific Review Board (Ref.: 2015/01158).

### 2.3. Data Analysis

Descriptive analysis was employed to profile the sample population. The values were expressed as percentage under the specific category and mean/median values are indicated where necessary. The age of onset across substance categories were analysed using Independent samples Kruskal-Wallis Test and the relationship between selected variables were studied using Pearson correlation. Multiple Linear regression was done to determine the effect of sociodemographic factors in the outcome of interest (years to death after the onset of substance use, days to death since last visit to the clinic). The model considered gender, ethnicity, employment status, marital status and comorbidities (DSM-IV Axis I). The data was analysed using IBM SPSS statistics version 23 (IBM, Armonk, NY, USA).

## 3. Results

### 3.1. Demographics

Among the substance users, 90.5% (*n* = 38) were males and 9.5% (*n* = 4) females (Table 1). All of them were Singapore citizens. 

The majority (*n* = 29; 69%) were Chinese, followed by Indians (*n* = 7; 16.7%) and Malays (*n* = 6; 14.3%). About half of them were married (*n* = 17; 40.5%), 38.1% (*n* = 16) were single, 19% (*n* = 8) divorced and 2.4% (*n* = 1) separated. A large proportion of the subjects lived with their family of origin (*n* = 19; 46.3%) and were employed (*n* = 17; 42.5%). The mean age at death was 44.9 (SD ± 13.1, age range 18–69).

### 3.2. Substance Use among the Subjects and Comorbidities

Among those who used substances, 33.3% (*n* = 14) used alcohol (based on the active DSM-IV diagnosis and supported by urine tests/breath analyser), and the remainder used other psychoactive substances. Alcohol dependence was present in 23.8% (*n* = 10) of cases and alcohol abuse was 7.1% (*n* = 3). Among the 10 subjects with alcohol dependence, one subject was dependent on multiple substances and was therefore grouped under substance use with more than two substances (Table 2). The majority of the subjects had a diagnosis of opioids (*n* = 20; 47.6%) or benzodiazepines dependence (31%, *n* = 13), among which eight subjects were dependent on both opioid and benzodiazepine. These subjects were included under the opioid and benzodiazepine group. Amphetamine and solvent use contributed to 2.4% (*n* = 1) each and cases with more than two substances were grouped as “substance use with more than two substances” and they contributed to 7.2% (*n* = 3, Table 2). For the purpose of the descriptive analysis, subjects were grouped into six groups: (1) Alcohol alone (dependence, abuse and use) (2) Opioid alone (dependence) (3) Benzodiazepine alone (dependence) (4) Opioid and Benzodiazepine together (5) Substance dependence involving more than two substances which includes polysubstance dependence with alcohol and without alcohol (6) others (includes amphetamine and solvent abuse).

Approximately 31% (*n* = 31) of the sample had Axis I psychiatric comorbidities. Seven subjects (16.6%) with alcohol use (abuse or dependence) had psychiatric comorbidities compared to three subjects with opioid dependence. Other illnesses such as depression (*n* = 2), anxiety (*n* = 1), schizophrenia (*n* = 1), psychosis (*n* = 1), gambling (*n* = 2) and behavioral problems (*n* = 1) were observed in trivial proportions. Approximately 16.7% had an Axis II diagnosis of personality disorder and 57.1% (*n* = 24) had an Axis III diagnosis: hepatitis C (*n* = 6; 14.3%), hypertension (*n* = 5; 11.9%), asthma (*n* = 4; 9.5%) and cardiovascular conditions (*n* = 3; 7.1 %) were the most frequent illnesses. Majority (*n* = 36; 85.7%) had psychosocial problems (Axis IV). Global Assessment of Functioning score (GAF, Axis V) was recorded for 31% (*n* = 13) of the subjects and it showed a mean score of 62.3 (SD ± 6). A detailed description of various Axis IV conditions can be found in Table 3.

### 3.3. Substance Use Profile and Manner of Death

Among the substances reported (self-reported by the subject, not supported by urine tests or diagnostic criteria) opioids, benzodiazepines and alcohol were the most abused drugs (Table 4). The age of onset for the substance use ranged from 11 to 59 years, with a mean age of 30 (± 11), 32.7 (± 12), and 22.8 (± 12) for opioid, benzodiazepines and alcohol respectively. Kruskal-Wallis test was conducted to examine the distribution of age of onset of substance across different categories of substances. No statistically significant differences (χ^2^ (2) = 5.9, *p* = 0.05) were seen in the age of onset between different substance types. Heroin was the most abused drug among the opioid group (54.4%, *n* = 12) followed by buprenorphine (*n* = 5). The majority of the opioid users were daily users (88.9%, *n* = 16) with an average use on 28.2 days (mode: 30, median: 30). Among the benzodiazepine users, midazolam (dormicum) was the most abused benzodiazepine (68.4%, *n* = 13) with an average monthly use of 22.8 days (*n* = 12, mode: 30, median: 30). Subjects who used alcohol reported an average monthly use of 19.6 days (mode 30 days, median 20).

History of polysubstance use was reported by 65.9% (*n* = 27; 65.9%). Heroin (*n* = 17; 63%), cannabis (*n* = 9; 33.3%), buprenorphine (*n* = 11; 40.7%) and midazolam (*n* = 8; 29.6 %) were the commonly used drugs reported by polysubstance users (Table 4).

Of the total number of unnatural deaths during the given period, suicide formed a large proportion (*n* = 27; 64.3%) followed by accidental death (*n* = 15; 35.7%). Among the accident cases, seven (16.7%) were due to accidental overdose. The substances used by the suicide group included alcohol (33.3%, *n* = 9), opioid (25.9%, *n* = 7) and opioid-benzodiazepine combinations (14.8, *n* = 4). Opioid was the most abused drug in accidental deaths (*n* = 5; 33.3%) followed by alcohol and opiate-benzodiazepine combination (*n* = 4; 26.7% each, Table 5). Intentional fall from high-rise buildings was the common mode of suicide in 85% (*n* = 23) of the cases.

The mean age for accidental deaths and completed suicides were 44.4 and 45.1 years, with a median of 47 and 44 years respectively. The mean and median years to death after the onset of substance use was 19.4 and 17 years for accidental deaths and 16.3 and 10.5 years for suicides respectively.

Notably, when measured from their last visits, the mean years at death for accidents and suicides were 1.07 and 2.2 years, respectively. In both cases the median years were 0, which indicates that the death had occurred within 12 months from their last visit to NAMS clinic. More than half of the cases (*n* = 24, 57.1 %) had died within the first year from their last visit to the clinic. The remaining deaths had occurred between wider ranges (2 to 17 years) after the last visit to NAMS clinics. For those deaths that had occurred within the first year from the last appointment, 12 deaths occurred within 30 days of their last visit. Of this, six deaths had occurred within 7 days of their last visit. Suicide was the common cause of death (*n* = 9) among those who died within the 30 days of their last appointment.

Among those who sought admission to the hospital, 15 had undergone inpatient detoxification programme for their substance abuse related difficulties and 23 were admitted for other psychiatric conditions. The mean number of admissions to inpatient detoxification programme and other general psychiatric wards were 0.8 (SD ± 1.4) and 2.2 (SD ± 3.5) respectively, which showed that the inpatient admissions were predominantly for general psychiatric conditions than for substance abuse related conditions.

Pearson’s correlation was employed to determine the relationships between the collected variables. There was a moderate negative correlations between the age of onset of the main substance and years to death (rp(35) = −0.48, *p* = 0.004), total number of clinic sessions defaulted and time to death after the last visit (rp(39) = −0.35, *p* = 0.029), and number of admissions to general psychiatric ward and time to death after the last visit (rp(41) = −0.34, *p* = 0.032). After controlling for the sociodemographic factors, a strong negative correlation was observed between years to death after the onset of substance use and age of onset of substance use. Gender, marital status, employment and age of onset were significant predictors of years to death after the onset of substance use (Supplementary Tables S1–S3). Females tend to have a significantly higher chance to die early when compared to males (B = −20.4, CI: −36.8–3.9, *p* = 0.02). Marriage (B = 11.9, CI: 2.9−20.9, *p* = 0.01) and employment (B = 8.5, CI = 0.76–16.3, *p* = 0.03) were protective against early death when compared to those who were single and unemployed. Ethnicity and employment status were stronger predictors of unnatural death following the last visit to the clinic than the number of defaulted clinic sessions and admissions to general psychiatric wards. Compared to Malays, Chinese (B = −959.63, CI: −1743.2–176.1, *p* = 0.02) and Indians (B = −1209.8, CI: −2181.5–238.1, *p* = 0.02) tend to succumb to unnatural death early after their last visit to the clinic. Employment was protective against early death (B = 511.3, CI: 5.5–1017.2, *p* = 0.04).

### 3.4. Past Psychiatric History and Suicidality

Of the total cases (*n* = 41), 63.4% (*n* = 26) had a history of mental illness. Depressive disorder (53.8%), was the common diagnosis among those with a reported history, followed by polysubstance use (26.9%), adjustment disorder (23.1%) and psychosis (substance induced, 15.4%). A history of adverse childhood experiences was captured in 32 cases, out of which 28.1% (*n* = 9) reported adversities such as childhood abuse (*n* = 3), involvement in gangs (*n* = 3), truancy (*n* = 2) and bullying (*n* = 1). Seven cases (20.6%) had a family history of mental illness and eight (27.6%) had a family history of addiction. Half of the cases (*n* = 21) had presented with past history of suicidal ideations. Of those who presented with past history of attempted suicides 34.1% (*n* = 14), 10 had eventually committed suicide. A history of non-suicidal self-harm was recorded for 33.3% (*n* = 13) of the subjects. Among the 27 who committed suicide, 40% had a history of non-suicidal self-harm; while 55.6% had a history of suicide ideations and 38.5% had attempted suicide.

### 3.5. History of Drug Related Offences

Among the 42 cases, 76% (*n* = 32) reported forensic history, out of which 57.1% (*n* = 24) had history of being imprisoned for drug related offences. The detailed description of forensic history and substance type is depicted in Table 6. Almost 60% (*n* = 18) of those with forensic history committed suicide.

## 4. Discussion

Singapore has an overall crude death rate of 4.8 per 1000 residents [30]. The average death rate reported during the study period was 4.7 per 1000 residents, of which a suicide rate of 8.4 per 100,000 residents [31] had stayed relatively stable over the years. In our retrospective analysis, a higher proportion of suicides (64.3%) compared to other unnatural causes was observed. Caution must be exerted while interpreting our findings as these results cannot be generalised to all substance users, as the datasets only comprise coroner’s unnatural death enquiries for those who had sought treatment with NAMS. Highest number of suicides was associated with clients who used opioids (those who used opioids alone or in combination with benzodiazepine) followed by alcohol and benzodiazepines users. Many studies have previously explored the common substances implicated in unnatural death and alcohol topped the list [32]. Contrary to that, Gossop et al., [33] observed opioids as the most common drugs in post-mortem examination of substance users, followed by benzodiazepines and then alcohol. We observed a higher proportion of opioids users followed by alcohol and then benzodiazepines, indicating a distinct pattern that is probably region specific. This can be explained by the fact that opioid dependence runs a chronic course along with significant psychosocial, legal and physical complications. Comorbidities such as mood and anxiety disorders which can further increase the risk of suicide. A direct comparison of our data against larger studies is restricted due to the small sample size of the current study.

Among the cases studied, suicide was the main contributor (64.3%) to unnatural death followed by accidental causes. There were no homicides reported among the cases. Similar trends have been observed in a national cohort in Taiwan [7]. A comparison of the Taiwanese cohort with US showed that the suicide rate is 20 times higher in Taiwan, which was attributed to higher severity of drug use and co-morbid conditions. Severity of drug use was not recorded in our study, nonetheless the higher rate of Axis I disorders supports the above observation. Depression is a risk factor for suicide [34]. Though more than half of the cases had reported a past history of depression, the number of patients who had an active diagnosis of clinical depression was low in our cohort. Nonetheless, this may not reflect the true prevalence rate, as many of our opioid and alcohol users do not stay long enough in our inpatient treatment program to complete the assessment of comorbidities. Suominen and colleagues [35] in their 5-year follow up study observed similar trends in unnatural death where suicide formed half of the reported deaths among substance users. Accidental overdoses are higher among drug users in western countries [36], but in our data, suicide is higher than drug overdose related deaths. Although the reasons for this observation are not very clear, we assume that this may be partly due to decreasing intravenous drug use among opiate users in Singapore based on our clinical data, low purity of diamorphine (4%) available in street heroin [37] and generally low prevalence rate for opioid prescription abuse in Singapore.

Opioids (heroin) and alcohol were the two main drugs associated with suicide among the study population. Alcohol use and suicide among patients with alcohol use disorders are highly prevalent and linked. Studies have shown a higher risk of suicide among those with alcohol use disorder [38]. The underlying factors for suicide among this population are multifactorial, involving social and biological elements. Psychiatric comorbidities are identified by many studies as a factor that contributes to these suicides [39]. Our sample showed 53.8% of those with alcohol abuse/dependence had Axis I comorbidity. A study conducted in opioid abusers who underwent treatment showed a suicide attempt rate of 17.3%, which is four times higher than community sample. Moreover, those who attempted suicide had childhood adverse events, psychiatric comorbidities along with poorer social/ psychological functioning [40,41]. Similar observations were recorded in a Finnish study [35] among drug users, where self-harm increased the risk of suicide by 40-fold and male gender was identified as one of the risk factors. A comparable observation was recorded in our study albeit, the sample size and analysis limitations limit the generalisation of these observations.

The current study indicated that almost half of the cases had a forensic history and majority of them had committed suicide. Research has shown that early and repeated offences, especially drug related offences are linked to high mortality risk [42]. In Singapore, drug consumption and possession are chargeable offences under the zero-drug tolerance policy and so it is not surprising that many get arrested and taken into prison where they undergo lengthy rehabilitation programs before they are released back into the community. Ministry of Home Affairs extend their support for drug offenders after their release from prison in the form of providing jobs, accommodation and psychological support through Singapore Corporation of Rehabilitative Enterprises. Nevertheless, substance use disorder is a chronic relapsing and remitting illness and so the frequent relapses that lead to subsequent psychosocial disadvantages, frequent clashes with criminal justice system and poor overall engagement with treatment services could all contribute to increased mortality.

This study has several limitations although it is the first of its kind from Singapore involving addiction patients. The unnatural deaths enquiries received from the coroner are not inclusive of all deaths among those with substance use disorder. They include deaths that are classified as “unnatural” where the deceased had previous contact with NAMS treatment service. Hence this study represents only the treatment seeking population from NAMS. We were also unable to ascertain the impact of the duration of their prison sentences, the type and quality of community-based rehabilitation services attended and the precise details of comorbid physical health conditions on increasing the risk for unnatural deaths due to lack of data on such factors. Our study did not have a matched comparison group therefore is subject to observational bias. The small sample size of the study is a major hurdle in comparing the results against larger cohort studies and drawing strong conclusions. A prospective study with a larger sample size and with robust analysis will be able to give a clearer picture of the drug use trajectory and unnatural death.

In Singapore, Central Narcotics Bureau’s drug situation report 2018 [43] informed that there was an 11% year-on-year increase in drug users arrested in 2018, primarily due to the enforcement efforts put in place to detect New Psychoactive Substances (NPS) such as synthetic cannabinoids and methamphetamine abusers. It is worth looking at the impact of new synthetic substances on our treatment seeking population and the evidence emerging that synthetic cannabinoids are reported to cause severe adverse events such as stroke, seizure, myocardial infarction, rhabdomyolysis, acute kidney injury, psychosis and associated deaths [44]). Another big concern for clinicians worldwide is the significant increases in overdose deaths reported in the US involving new synthetic opioids such fentanyl and others [45]. Therefore, future research will include exploring the role of these substances in unnatural deaths.

## 5. Conclusions

This is the first time we studied the psychosocial factors that may have an association with unnatural death in our clinical population. Despite the small sample size and number of other limitations in the study, our analysis suggests that previous contact with mental health service, early onset of substance use, number of defaulted clinic sessions are associated with unnatural death. The study also highlighted more unnatural deaths among alcohol and opioid users compared to other substance users and these deaths occurred within the first 12-month period after their last contact with our service. These findings align with other previous studies and reiterate the importance of early identification of risky substance users and the necessity of a close follow up.

## Figures and Tables

**Table 1:** Socio-demographic characteristics of the subjects.

Demographics of the Subjects *n* (%)	
Gender	*N* = 42
Males	38 (90.5)
females	4 (9.5)
Nationality	*N* = 41
Singaporean	100
Ethnicity	*N* = 42
Chinese	29 (69)
Malay	6 (14.3)
Indian	7 (16.7)
Religion	*N* = 37
Christianity	10 (27)
Buddhism	7 (18.9)
Hinduism	4 (10.8)
Islam	6 (16.2)
Taoism	2 (5.4)
Others	8 (21.6)
Marital status	*N* = 42
Single	16 (38.1)
Married	17 (40.5)
Separated	1 (2.4)
Divorced	8 (19)
Living arrangement	*N* = 41
Living alone	7 (17.1)
living with spouse or children	9 (22)
Living with family of origin (e.g., parents, sibling)	19 (46.3)
Others	6 (14.6)
Employment	*N* = 40
Employed	17 (42.5)
Unemployed	23 (57.5)

**Table 2:** Represents the percentage of subjects using various drugs (based on diagnosis).

Category	Dependence	Abuse	Use
	*n* (%)	*n* (%)	*n* (%)
Alcohol alone	9 (21.4)	3 (7.1)	1 (2.4)
Opioid alone	12 (28.6)		
Benzodiazepine alone	4 (9.5)		
Opioid and Benzodiazepine	8 (19.0)		
Substance dependence with more than two substances			
Polysubstance dependence without alcohol	2 (4.8)		
Polysubstance dependence with alcohol dependence	1 (2.4)		
Others			
Amphetamine		1 (2.4)	
Solvent		1 (2.4)	

**Table 3:** DSM-IV Axis IV diagnosis among the study subjects.

Type of Problem	*n* (%)
Employment related	18 (42.9)
Financial	4 (9.5)
Interpersonal relationship	10 (23.8)
Accommodation	4 (9.5)
Family related	4 (9.5)
Marital	9 (21.4)
Legal	9 (21.4)
Others	1 (2.4)

**Table 4:** Substance use profile (self-reported).

Substance	*n* (%)	Age of Onset	
Alcohol	15 (35.7)	22.8 (± 12)	
Opioid	22 (52.4)	30 (± 11)	
Benzodiazepines	19 (45.2)	32.7 (± 12)	
Methamphetamines	3 (7.1)	22.3 (± 6.1)	
Cannabis	3 (7.1)	19.5 (± 2.1)	
Ketamine	1 (2.4)	21	
Inhalants	1 (2.4)	11	
Polysubstance *n* (%)			
Heroin	17 (63)	Midazolam	8 (29.6)
Opium	2 (7.4)	Diazepam	2 (7.4)
Buprenorphine	11 (40.7)	Lorazepam	2 (7.4)
Methamphetamines	5 (18.5)	Nimetazepam	6 (22.2)
Cannabis	9 (33.3)	Benzodiazepines NOS	2 (7.4)
Codeine	5 (18.5)	Tramadol	1 (3.7)
Cough Mixtures	3 (11.1)	Inhalant	2 (7.4)
Ecstasy	4 (14.8)	LSD	2 (7.4)
Ketamine	4 (14.8)	Alprazolam	5 (18.5)
Nitrazepam	2 (7.4)		

NOS: Not otherwise specified; LSD: Lysergic acid diethylamide.

**Table 5:** Substance use and mode of death.

Cause	Alcohol *n* (%)	Opioids *n* (%)	Benzodiaze-pine *n* (%)	Opioids and Benzodiazepines *n* (%)	Substance Use with More than 2 Substances *n* (%)	Others *n* (%)
Accidental death (*N* = 15)	4 (26.7)	5 (33.3)	1 (6.7)	4 (26.7)	1 (6.7)	0
Suicide (*N* = 27)	9 (33.3)	7 (25.9)	3 (11.1)	4 (14.8)	2 (7.4)	2 (7.4)

**Table 6:** Forensic history of the subjects.

History	Alcohol *n* (%)	Opioids *n* (%)	Benzodiazepine *n* (%)	Opioids and Benzodiazepines *n* (%)	Abuse/Dependence Involving More than Two Substances *n* (%)	Others *n* (%)
Forensic history alone (*N* = 30)	9 (30)	9 (30)	3 (10)	5 (16.7)	2 (6.7)	2 (6.7)
Drug offences alone (*N* = 24)	4 (16.7)	8 (33.3)	2 (8.3)	6 (25)	2 (8.3)	2 (8.3)
Both (*N* = 22)	4 (18.2)	7 (31.8)	2 (9.1)	5 (22.7)	2 (9.1)	2 (9.1)

## Supplementary Materials

The following are available online at https://www.mdpi.com/1660-4601/16/15/2743/s1, Table S1: Predictors of years to death, Table S2: Predictors of days to death following last visit to the addiction clinic, Table S3: Predictors of days to death since last visit following inpatient admissions.
