# Supplementary material for: Unnatural Death among Treatment Seeking Substance Users in Singapore: A Retrospective Study
Source: Int J Environ Res Public Health. 2019 Jul 31;16(15):2743. doi: 10.3390/ijerph16152743 (PMC6695629; doi:10.3390/ijerph16152743)
Supplement: Supplementary file 1 [file ijerph-16-02743-s001.pdf]

Supplementary Table 1. Predictors of years to death

Parameter	B	Std. Error	t	Sig.	95% Confidence Interval	
					Lower Bound	Upper Bound
**comorbidity**						
Yes	-6.4	3.9	-1.6	.12	-14.6	1.8
No	0[a]	.	.	.	.	.
**Gender**						
Female	-20.4	7.9	-2.6	.02	-36.8	-3.9
Male	0[a]	.	.	.	.	.
**Ethnicity**						
Chinese	10.9	5.9	1.8	.08	-1.4	23.1
Indian	-1.4	7.1	-.2	.85	-16.1	13.2
Malay	0[a]	.	.	.	.	.
**Marital status**						
Divorced	7.2	4.9	1.5	.16	-2.9	17.4
Married	11.9	4.3	2.7	.01	2.9	20.9
Separated	16.9	13.3	1.3	.22	-10.6	44.5
Single	0[a]	.	.	.	.	.
**Employment Status**						
Yes	8.5	3.8	2.3	.03	.8	16.3
No	0[a]					
Age of onset	-.8	.2	-4.6	.000	-1.2	-.5

Supplementary Table 2. Predictors of days to death following last visit to the addiction clinic

Parameter	B	Std. Error	t	Sig.	95% Confidence Interval	
					Lower Bound	Upper Bound
**comorbidity**						
Yes	16.5	272.8	.06	.95	-542.2	575.2
No	0[a]	.	.	.	.	.
**Gender**						
Female	-792.9	457.5	-1.7	.09	-1730.1	144.1
Male	0[a]	.	.	.	.	.
**Ethnicity**						
Chinese	-959.6	382.5	-2.5	.02	-1743.2	-176.1
Indian	-1209.8	474.4	-2.6	.02	-2181.5	-238.1
Malay	0[a]	.	.	.	.	.
**Marital status**						
Divorced	282.8	335.5	.8	.41	-404.5	970.0
Married	218.0	281.0	.8	.44	-357.6	793.7
Separated	1391.9	894.7	1. 6	.13	-440. 8	3224.7
Single	0[a]	.	.	.	.	.
**Employment Status**						
Yes	511.3	246.9	2. 1	.048	5.5	1017.2
No	0[a]					
Defaulted clinic sessions	-35.9	20.79	-1.79	.094	-78.39	6.6

Supplementary Table 3. Predictors of days to death since last visit following inpatient admissions

Parameter	B	Std. Error	t	Sig.	95% Confidence Interval	
					Lower Bound	Upper Bound
**comorbidity**						
Yes	-289.8	451.1	-.6	.53	-1211.1	631.6
No	0[a]	.	.	.	.	.
**Gender**						
Female	-1164.9	788.1	-1.5	.15	-2774.5	444.7
Male	0[a]	.	.	.	.	.
**Ethnicity**						
Chinese	-157.3	620.5	-.25	.80	-1424.5	1109.9
Indian	-694.7	754.2	-.9	.36	-2234.9	845.6
Malay	0[a]	.	.	.	.	.
**Marital status**						
Divorced	-271.8	563.162	-.48	.63	-1421.9	878.3
Married	-165.5	478.0	-.35	.73	-1141.7	810.8
Separated	1696.1	1543.8	1.1	.28	-1456.7	4848.9
Single	0[a]	.	.	.	.	.
**Employment Status**						
Yes	136.9	413.4	.33	.74	-707.4	981.2
No	0[a]					
Admission to the hospital	-105.9	59.5	-1.7	.09	-227.5	15.6
